# Contrast agents for photoacoustic imaging: a review of stem cell tracking

**DOI:** 10.1186/s13287-021-02576-3

**Published:** 2021-09-25

**Authors:** Soorya James, Kai Neuhaus, Mary Murphy, Martin Leahy

**Affiliations:** 1 Tissue Optics and Microcirculation Imaging facility,School of Physics, National University of Ireland, Galway, University Road, Galway, Ireland; 2 The Regenerative Medicine Institute, National University of Ireland, Galway, University Road, Galway, Ireland; 3 ICFO-Institut de Ciències Fotòniques, The Barcelona Institute of Science and Technology, Castelldefels, Spain

**Keywords:** Photoacoustic, Optoacoustic, Stem cell tracking, Mescenchymal stem cells, Contrast agents

## Abstract

With the advent of stem cell therapy for spinal cord injuries, stroke, burns, macular degeneration, heart diseases, diabetes, rheumatoid arthritis and osteoarthritis; the need to track the survival, migration pathways, spatial destination and differentiation of transplanted stem cells in a clinical setting has gained increased relevance. Indeed, getting regulatory approval to use these therapies in the clinic depends on biodistribution studies. Although optoacoustic imaging (OAI) or photoacoustic imaging can detect functional information of cell activities in real-time, the selection and application of suitable contrast agents is essential to achieve optimal sensitivity and contrast for sensing at clinically relevant depths and can even provide information about molecular activity. This review explores OAI methodologies in conjunction with the specific application of exogenous contrast agents in comparison to other imaging modalities and describes the properties of exogenous contrast agents for quantitative and qualitative monitoring of stem cells. Specific characteristics such as biocompatibility, the absorption coefficient, and surface functionalization are compared and how the labelling efficiency translates to both short and long-term visualization of mesenchymal stem cells is explored. An overview of novel properties of recently developed optoacoustic contrast agents and their capability to detect disease and recovery progression in clinical settings is provided which includes newly developed exogenous contrast agents to monitor stem cells in real-time for multimodal sensing.

## Introduction

Stem cell therapies have shown immense potential for treating various degenerative diseases in pre-clinical settings and clinical trials. However, for the successful quantitative and qualitative monitoring of stem cells and the understanding of their regenerative mechanisms and pathways, stem cell labelling and tracking are crucial. Differentiating cells of interest from the host cells using contrast agents and visualising the in vivo distribution and migration to the targeted site using suitable imaging modalities is of utmost importance from a therapeutic point of view. Determining the location and quantity of the cells, monitoring the delivery of cells to the target tissues and their long-term fate and biodistribution is possible with available imaging modalities [1, 2].

The advantages and limitations of currently available imaging modalities for stem cell tracking applications has been extensively reviewed [3–5]. Among these, MRI is widely used for stem cell tracking due to its ability for long-term cell tracking and high spatial resolution. However, it cannot be used for real-time imaging due to its poor temporal resolution, measured in minutes, and low sensitivity which demands high doses of contrast agents [1, 2]. Labelling stem cells with a high concentration of iron oxide contrast agents could also lead to a reduction and distortion in their differentiation potential, which can further affect the therapeutic effects of stem cells [6, 7]. Radioactive imaging consisting of single-photon emission computed tomography and positron emission tomography are sensitive techniques but lack spatial resolution and do not allow for long-term imaging of cells due to the short half-lives of radioisotopes used. While reporter gene labelling can be utilised for MRI and radioactive imaging, it still has a limited clinical presence because of cell safety issues such as immunogenic responses [1, 2]. Computed tomography has sufficient temporal resolution but has limited sensitivity, poor soft-tissue contrast and utilises ionising irradiation, which can affect the cell biology [8–10]. Fluorescence and bioluminescence imaging are optical imaging methods with high sensitivity and high resolution. However, they are challenging to use for clinical applications due to optical scattering that results in shallow penetration depth, and phototoxicity of the imaging dyes used [1, 2, 11, 12].

**Fig. 1:**
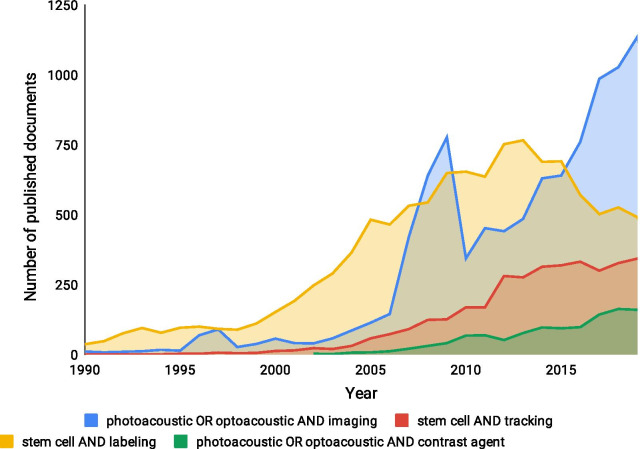
Graph depicting the number of publications using the various search terms as shown. (Source: Scopus)

Photoacoustic imaging (PAI) or optoacoustic imaging (OAI) has been gaining popularity since 1990 with the increase in their significance to different biomedical imaging applications. With the advent of stem cell therapy, the interest in stem cell labelling seemed to increase in parallel with OAI, although the trend shows a dip around 2012 (Fig. 1). This may be due to limitations in the development of contrast agents. With the development of optoacoustic contrast agents and advancements in the imaging infrastructure, research on stem cell labelling and tracking using contrast agents continues to be of relevance. This could be due to the growing interest in optimising contrast agents for clinical applications to gain a better understanding and design of stem cell therapies. However, there is still a need to improve the sensitivity of imaging methods and design multifunctional contrast agents for specifically labelling stem cells. OAI methodologies are steadily evolving and have proven to be successful in both preclinical and clinical applications. OAI is a non-invasive approach and can generate high resolution images of biological tissues at greater penetration depths. Due to its real-time imaging capability, OAI can be utilised as a significant tool to improve stem cell therapies as it can quantify the number of cells that is being delivered to the site of interest. Moreover, OAI can monitor the migration of the delivered cells for prolonged periods and to observe the progress of cell engraftment and responses of the local tissues to the therapy [13–17]. This article reviews the strengths and shortcomings of various optoacoustic contrast agents and discusses the essential parameters for engineering exogenous contrast agents. Special attention is paid to the development of multimodal contrast agents that are relevant in clinical settings, are biocompatible, have low toxicity, and can enhance the imaging resolution enabling tracking of stem cells.

## Optoacoustic Imaging

Optoacoustic imaging (OAI), often referred to as photoacoustic imaging is based on the photoacoustic effect, which is the conversion of pulsed light into ultrasound waves in optically absorbing samples. OAI is carried out by illuminating a sample with an intense nanosecond pulsed laser source. This causes the photons to propagate and diffuse through the tissue. Optically absorbing molecules absorb some of this light, which is then partially converted to heat. This results in a rapid thermoexpansion and contraction in the tissue, which generates a pressure wave. An ultrasonic transducer detects this pressure wave, and subsequent digital processing converts the ultrasonic signal into images revealing molecular and structural properties of the sample. OAI utilises either an ultrasound detector array or a single scanned detector and an inverse algorithm to reconstruct cross-sectional or three-dimensional images of the sample, which can be a biological specimen or even human tissue. The acoustic pressure waves are proportional to the absorption coefficient of the medium being irradiated $\mu _a$, the fluence of the irradiated energy ${\varPhi }$ (light energy per meter squared), and the Grüneisen coefficient of tissue ${\Gamma }$. Thus, the maximum initial pressure $P_0$ which predicts the pressure wave at the absorber of interest is given by Eq. 1:

1 $$\begin{aligned} P_0 = \mu _a {\varPhi } {\Gamma } \end{aligned}$$

**Fig. 2:**
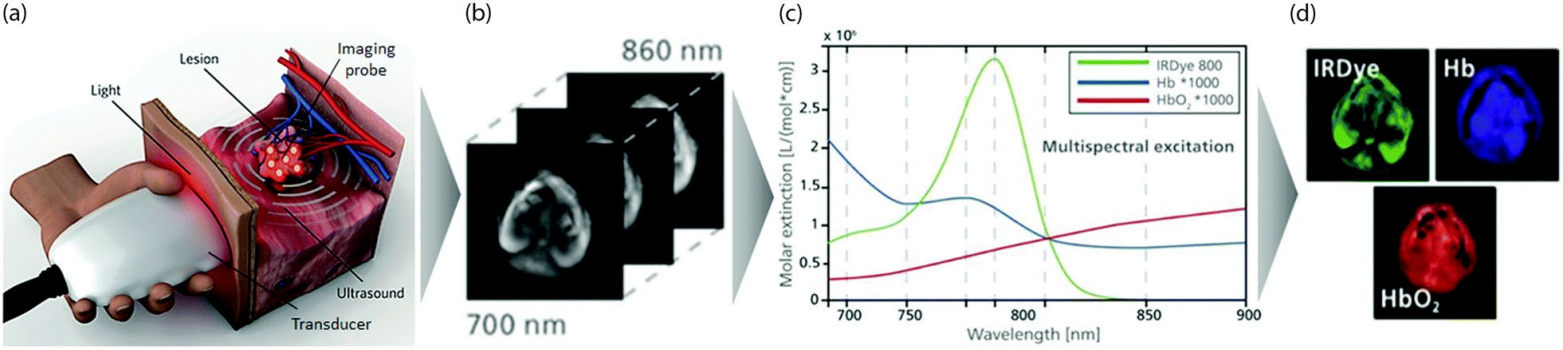
Principle of MSOT: **a** A handheld optoacoustic imaging system uses a light source to generate optoacoustic signals. **b** Optoacoustic responses after irradiating the target tissue with multiple wavelengths are collected and undergoes image reconstruction. **c** Spectral unmixing algorithms identify and differentiate the spectral contributions of endogenous photo absorbing molecules in the target tissue (e.g. oxy- or deoxygenated haemoglobin) from exogenous probes (e.g. IR Dye 800). **d** Separate images corresponding to each endogenous and exogenous chromophore is analyzed. Adapted with permission from [19]. Copyright $\copyright$ 2017, Royal Society of Chemistry

Multispectral Optoacoustic Tomography (MSOT) is a novel variant of functional OAI, which is currently receiving considerable interest. MSOT uses a broader range of excitation wavelengths that allows acquisition of signals from multiple endogenous chromophores like oxygenated and deoxygenated blood, exogenous chromophores such as nanoparticles and dyes simultaneously in vivo (Fig. 2) [18, 19]. Due to their intrinsic optical absorption properties, both oxygenated and deoxygenated hemoglobin have dominant absorption from the visible to near-infrared-I (NIR-I) range in comparison with surrounding water, whose optical absorption is only dominant in the farther infrared regions. Detecting the changes in oxygen saturation in blood vessels using optoacoustic imaging can be used to diagnose and study various pathological conditions in the body. However, the optical fluence arriving at various parts of the tissue is dependent on the light path and so is unknown in typical applications. A solution, proposed by Leahy [20], is to use the known absorption in arterial blood and interpolate between arteries to map fluence throughout the tissue volume of interest. In this way a calibrated quantification of oxygen saturation can be made, independent of the light transport properties of the tissue. This can also be applied to the concentration of contrast agents and the labelled substance such as stem cells. Melanin, on the other hand, has an intrinsic ability to absorb light over a broad range of the visible and near infrared-region, with its contrast decreasing at longer wavelengths. Melanin is mainly used as an endogenous contrast agent in OAI to study the progression of melanoma in patients and to track the metastatic growth at large penetration depths [21]. Since molecular detection becomes more sensitive and accurate using an increased number of wavelengths, multi-wavelength irradiation can improve quantitative volumetric imaging of molecular probes [22, 23]. MSOT can provide anatomical (whole body in small animals), functional, molecular, and kinetic information at high temporal and spatial resolution. Concerning stem cell tracking, MSOT can provide details of the inter- and intra-organ distribution of the administered cells at multiple wavelengths and can resolve the spectral signatures in each voxel imaged.

## Exogenous contrast agents

Exogenous contrast agents are utilised to improve the contrast between the region of interest and background, to detect structures that are otherwise difficult to target and to reach tissues at a greater depth. They aid in gathering information regarding a disease state, detection of biomarkers or biochemical and cellular processes with high sensitivity, specificity and signal-to-noise ratio (SNR). There is some flexibility when it comes to developing a contrast agent for OAI, as any molecule that absorbs light, efficiently transferring the energy to the surrounding tissue, causing a thermoelastic expansion in order to generate a pressure wave is a candidate [24]. Ideally, optoacoustic imaging probes should possess high absorption cross-sections and a superior ability to convert most of the absorbed light quickly into heat energy for efficient acoustic wave generation. Developing exogenous contrast agents that have a narrow and characteristic spectral profile that does not overlap with the endogenous contrast agents present in the body could be advantageous to separate them from the background at a tissue site using spectral unmixing algorithms. Apart from optical characteristics, various other parameters must be kept in mind for designing exogenous contrast agents for in vivo imaging and particularly stem cell tracking.

## Labelling of stem cells with exogenous contrast agents

Stem cells cannot be visualised directly by OAI as they do not have an adequate optical absorption coefficient; they must be labelled with efficient contrast agents. Before monitoring stem cells in a clinical setting, it is essential to optimize the labelling efficiency and investigate the cytotoxicity of contrast agents as they are highly dependent on the contrast agent composition, shape, size, concentration, and surface functional groups. Moreover, these factors indirectly affect the viability, differentiation, distribution, migration and engraftment of stem cells.

Adult stem cells such as mesenchymal stem cells (MSCs) are important because of their capability to maintain and repair their tissue of origin. MSCs can differentiate into tissue- specific cell types while maintaining their capacity for self-renewal, migrate to injured tissues naturally to promote vascularization [25] and immunomodulation to accelerate tissue regeneration [26, 27]. About 50% of the clinical trials have often utilized bone-marrow-derived MSCs [28]. MSCs have been isolated from many sources including fat obtained from liposuction procedures and bone-marrow, normally obtained from the iliac crest.

Studies have reported that less than 10% of administered stem cells have been successfully engrafted in target tissues, while the remainder were either trapped in reticulo-endothelial organs or underwent apoptosis shortly after implantation [29, 30]. Since direct labelling requires the cellular uptake of exogenous contrast agents before engraftment, there are limitations in the assessment of stem cell functionality in vivo due to dilution in the contrast agent upon proliferation. Indirect labelling is less preferred for clinical translation as it involves the incorporation of a reporter gene through efficient transduction of viral or transfection of non-viral vectors to ensure detection of the expressed reporters upon successful engraftment. This method also raises safety concerns with the possibility of viral vectors leading to mutagenesis, toxicity, immune and allergic responses if the expressed protein is secreted by the cells. As stem cells have the potential to repair and replace damaged tissue in the host organism, it is critical to observe if the cell labelling affects any regenerative properties.

Clinical translation from a pre-clinical setting for stem cell tracking applications critically requires monitoring of the spatial destination, the migration pathway and final distribution of the transplanted stem cells using non-invasive, longitudinal, and repetitive imaging. Engineering a contrast agent with desirable characteristics for stem cell tracking can monitor if the stem cells have reached the target tissue and survived after transplantation for efficient therapeutic activity. Table 1 provides an overview of the recent optoacoustic contrast agents used for stem cell tracking, their characteristics that allow them for efficient cellular uptake and long-term longitudinal monitoring in the NIR window and the number of minimum cells detected. The desirable characteristics for designing and developing an ideal exogenous contrast agent are discussed in more detail in the following sections.

**Table 1:** Examples of optoacoustic contrast agents used for in vivo/ex vivo stem cell tracking

Contrast agent in use	Surface Coating utilised	Opto-acoustic excitation wavelength (nm)	Intracellular concentration	Number of days of longitudinal tracking	Number of cells imaged	Imaging Modalities utilised	Stem cell-based application
ICG		810	0.25 mg/ml	3 days	3$\times 10^5$ cells	OAI	Cell transplantation based therapies [31]
PBP	Citrate	701	0.2 mg/ml	15 days	$10^6$ cells	OAI	Traumatic brain injury [40]
PBNP	Poly-L-lysine (PLL)	730	50 $\upmu$g/ml	14 days	5$\times 10^4$ cells	OAI	Ischemic brain damage [32]
AuNS		700	1.5 ng gold/cell		1000 cells/$\upmu$l	OAI	Neuro degenerative diseases [70]
		700	200 $\upmu$g gold/ml		5$\times 10^6$ cells	OAI	Stem cell delivery trabecular meshwork to treat glaucoma [49]
Gold nanotracer (Au NT)	PLL	700	[4.53$\pm$ 0.04]$\times 10^5$ Au NTs/cell	10 days	5$\times 10^4$ cells	OAI	Ischemic heart disease [42]
Iron oxide nanoparticle	PLGA	532, 700	20 $\upmu$g/ml			OAI	Delivery of resveratrol [33]
GNR	Silica	680	1.5$\times 10^6$ SiGNRs/cell	4 days	$10^5$ cells	OAI	Muscular dystrophy [37]
	Silica	760	3.5$\times 10^{-2}$ $\upmu$g Au/ml	15 days	2$\times 10^4$ cells	OAI	In vivo stem cell tracking [38]
	IR775c	790, 920	20 $\upmu$g/ml	10 days	$10^7$ cells/ml	OAI	Stem cell viability tracking [61]
OSPN$^+$	PLL	916, 1025	50 $\upmu$g/ml	14 days	$10^5$ cells	OAI	In vivo stem cell tracking [44]
PANP		705	4 nM		2000 cells	OAI	To track embryonic stem-cell derived cardiomyocytes [36]
SPIO@Au	Gold	810	4 $\upmu$g/ml	3 days	$10^6$ cells	OAI, MRI	MSC mediated brain lesion treatment [67]
AuNC		800	2.3$\times 10^4$ AuNCs/ cell		$10^5$ cells	OAI, Two-photon microscopy	In vivo stem cell tracking [51]
Gd(III)-SMNP		720–760	0.43 mg/ml		5$\times 10^5$ cells	OAI, MRI	In vivo stem cell tracking [68]
Iron oxide nanobubbles labelled with DiR	PLGA		240 $\upmu$g/ml	3 days	$10^5$ cells	US, OAI, MPI	Cardiac stem cell therapy [73]
Fe$^{3+}$-PEG-MNP			800 $\upmu$g/ml	35 days	2$\times 10^6$ cells	OAI, MRI	In vivo stem cell tracking [45]

## Desirable characteristics of optoacoustic contrast agents for stem cell tracking

For any biomedical and clinical studies that rely on stem cell tracking, it is crucial to design and develop exogenous contrast agents that can be produced reproducibly in large volumes. To ensure a prolonged half-life for in vivo longitudinal tracking in therapeutic studies, these contrast agents must be colloidally stable. Moreover, they must be biocompatible to avoid adverse immune responses and negative reactions in the vascular system, kidneys and blood-brain barrier. They should be designed with a surface that can be efficiently modified to bind to molecular probes like antibodies or peptides to be able to target specific receptors in cells and tissues of interest. For efficient optoacoustic tracking of stem cells, the exogenous contrast agents should be optimized to have a high light to acoustic signal conversion efficiency in the NIR region. Specifically, for stem cells, it is vital to ensure that the contrast agent does not affect the rate of proliferation and the differentiation potential of the cell. The surface charge of the contrast agent could also be perfected to facilitate higher cellular uptake, which directly translates to a higher contrast-to-noise ratio for effective imaging.

### Biocompatibility and colloidal stability

Biocompatibility defines a substance that does not disturb the biochemical process in living cells or organisms with no or minimal toxicity. Moreover, such substances should elicit no or minimal immunological response or minimal physiological reaction. A material is colloidally stable when it is resistant to changes in the chemical, physical or biochemical environment and does not physically disintegrate upon introduction of external stimuli. Upon cellular uptake of exogenous contrast agents after incubation in a biological medium, aggregation and agglomeration of contrast agents are induced because of their interaction with proteins. Biocompatibility and colloidal stability of the contrast agent can be improved by integrating or coating them with materials that make the surface of the contrast agent more neutral or hydrophilic.

**Fig. 3:**
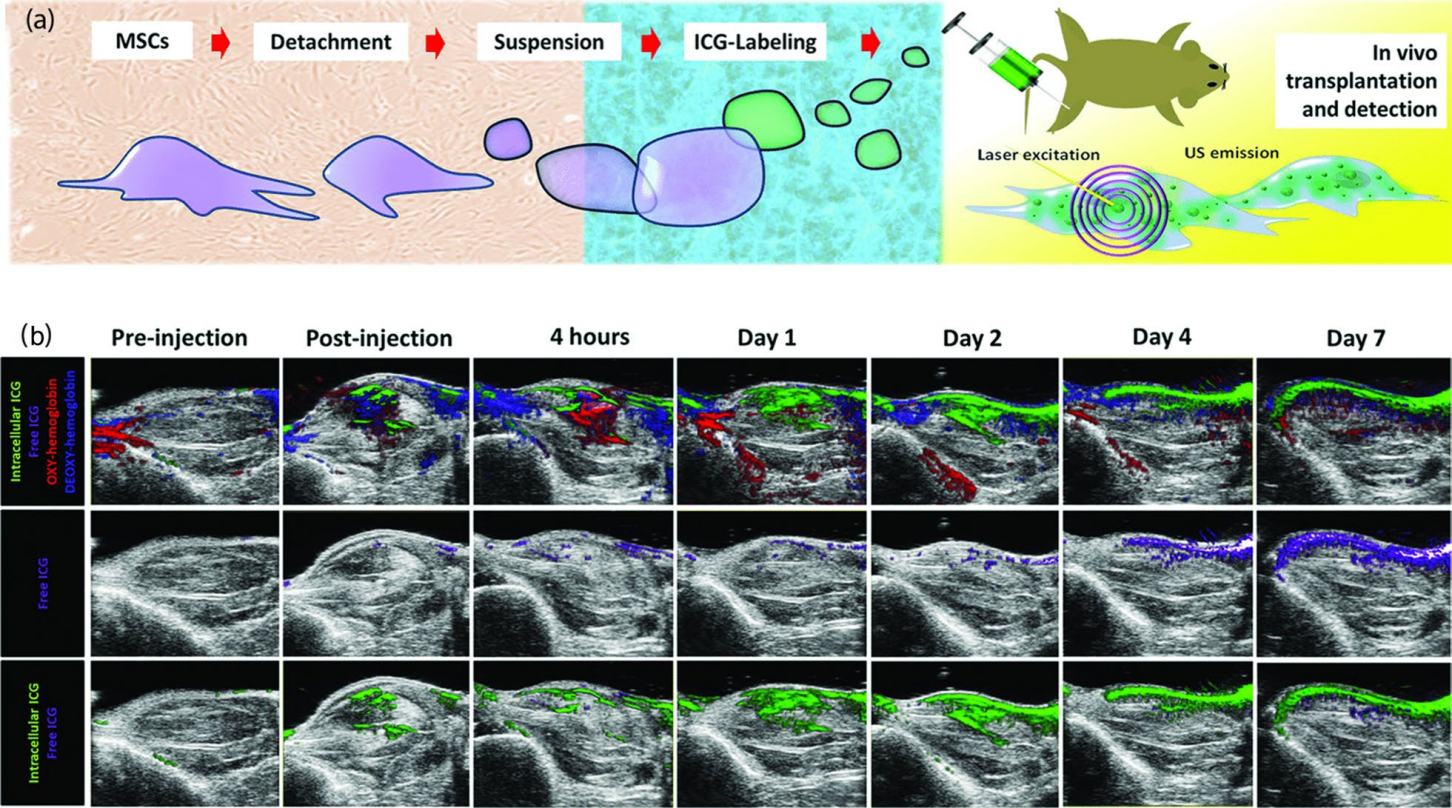
**a** ICG as an exogenous contrast agent for in vivo stem cell tracking in MSOT. **b** Representative OA monitoring of the cell engraftment over days (excitation wavelength: 810 nm) after spectral unmixing of signal in the right hindlimb. The free ICG-related signal is displayed in purple, intracellular ICG in green. ROIs were drawn on the muscular region (internal area of the hindlimb), excluding the unspecific signal generated by the skin-induced reflection artefacts (tissue depth $\le$ 1 mm).Adapted with permission from [31].Copyright $\copyright$ 2018, John Wiley and Sons

For optoacoustic contrast agents, the chemical structure of NIR dyes can be optimised for colloidal stability by introducing hydrophilic groups, triplet state quenchers and stabilising groups. Indocyanine Green (ICG) is a tricarbocyanine dye that has a 10% fluorescence quantum yield in water, thus able to release 90% of its excited non-radiative energy in the form of thermoelastic expansion, an advantage for optoacoustic imaging. Filippi et al. studied FDA approved ICG contrast agents to track MSC in vivo due to its excellent absorption properties and biocompatibility [31]. The study showed minimal toxicity per dose of ICG with an optoacoustic detection limit of 7,000 labelled cells (Fig. 3). Polymers such as poly-ethylene-glycol (PEG) and poly L-lysine (PLL) can be utilized to reduce the surface interaction of the contrast agent with the proteins and other small molecules present in the serum or blood. Prussian blue nanoparticles (PBNPs) coated with PLL were utilized by Kim et al. for optoacoustic stem-cell imaging [32]. The PLL coating enhanced the colloidal stability of PBNPs and facilitated easier cell internalisation, further contributing to a detection limit of 200 cells/$\upmu$l in vivo . Adjei et al. designed a multifunctional nanoparticle system which allowed delivery of resveratrol (RESV) intracellularly and enabled OAI of MSCs [33]. The stability of RESV-NPs was enhanced 18-fold due to the formulation of poly (lactic-co-glycolic) acid and iron oxide. Due to the enhanced colloidal stability of RESV-NPs, over 90% of the OA signal was retained even after the labelled MSCs underwent homogeneous differentiation.

For any type of biomedical application such as stem cell tracking, biocompatibility and colloidal stability of contrast agents are critical. Already available FDA-approved dyes like ICG which have a consistent intracellular uptake have been utilised for stem cell labelling and tracking. Coating nanoparticles with polymers can increase their colloidal stability for long periods of time at high ionic concentrations.OAI is a non-invasive and non-ionising biomedical imaging modality which is used for tracking stem cells, and it does not affect the proliferation and function of stem cells. On the other hand, contrast agents could have detrimental effects on functioning and differentiation potential of stem cells depending upon its chemical composition. Therefore, it is crucial to design OAI contrast agents which are biocompatible and non-toxic, and which do not have any undesirable effects on the proliferation, function, and safety of the labelled stem cells. Characterisation of contrast agent is critical to estimate the level of aggregation, change in surface charge and adsorption of proteins to the nanoparticle before and after interaction with biological media. Additionally, utmost care is taken to monitor the viability, toxicity and genetic stability of labelled cells in vitro before using these molecular probes for pre-clinical studies. For clinical applications, toxicology testing of the contrast agents on their own and in stem cells should be carried out under GLP conditions and in vivo (using mouse models) to assess any tumorigenic, hepatotoxic or neurotoxic potential [34]. The safety profile of a contrast agent can be defined after systematically investigating their biological interactions, toxicity, stability and retention both in vitro and in vivo.

### Enhancement in cellular uptake

The physicochemical properties such as the size, morphology and surface charge of contrast agents directly influence their cellular uptake mechanism and intracellular trafficking [35]. High therapeutic efficacy can be achieved by ensuring a safe and efficient entry of the contrast agents into stem cells as the route of entry affects the toxicity and biodistribution of the contrast agent. Cell-penetrating peptides (CPPs) can be utilized as inert vectors for delivery of cargo molecules like contrast agents due to their membrane-permeating properties and bioactive nature. Qin et al. utilised photoacoustic nanoparticles (PANPs) incorporating semiconducting polymers, where the PANP surfaces were encapsulated with a polymer lipid matrix with CPPs as labelling enhancers for tracking human embryonic stem-cell-derived cardiomyocytes (hESC-CMs) in living hearts [36]. Terminally differentiated cell types like hESC-CMs have limited endocytosis activity. Therefore, direct cellular labelling was replaced with the utilisation of CPPs which resulted in a detection sensitivity of 2000 cells.

**Fig. 4:**
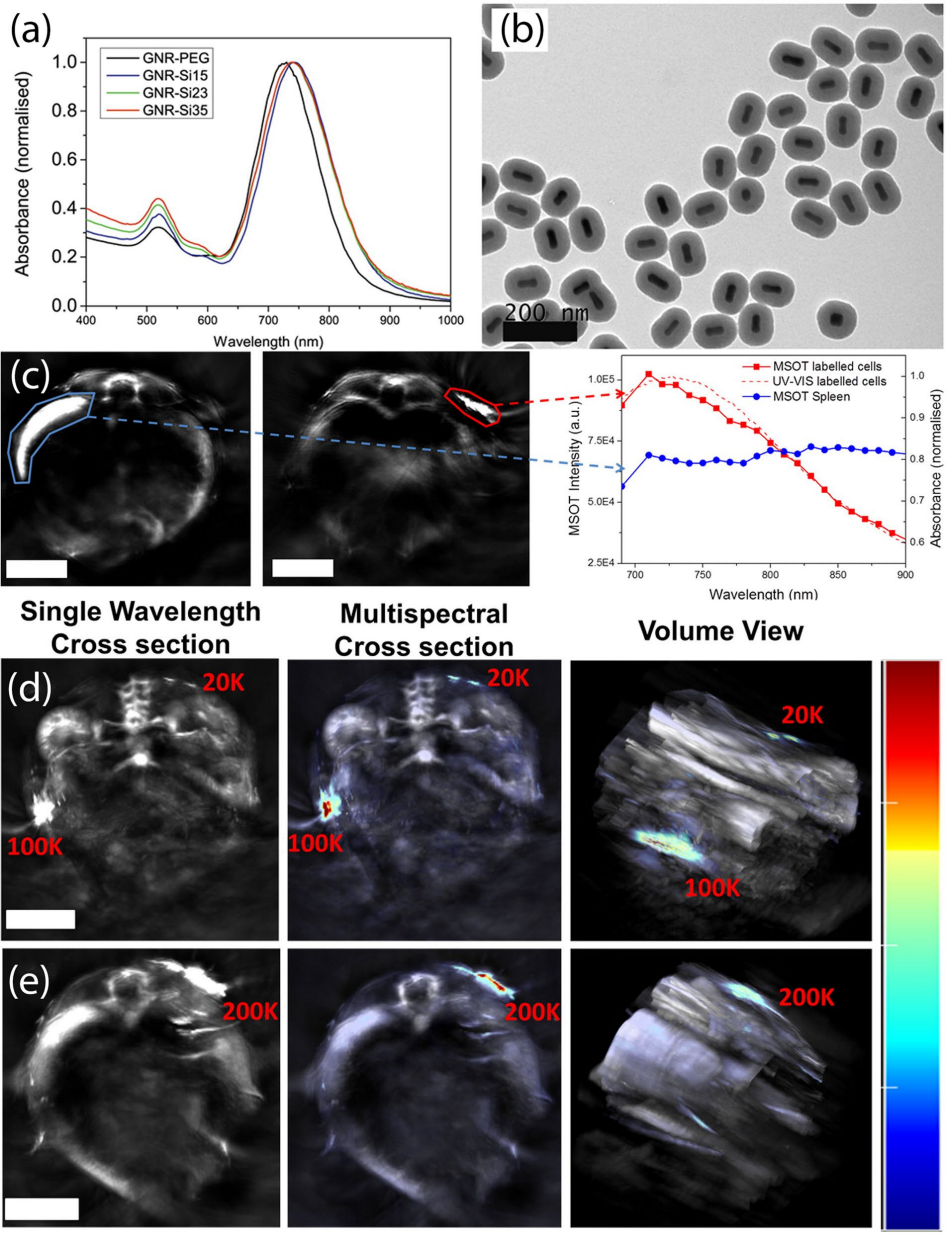
**a** Vis-NIR spectrum of GNRs before and after silica coating. **b** TEM pictures of GNRs coated 35 nm silica shells. **c** Cross section images of spleen (blue ROI) and GNR-Si35-labeled cells (red ROI). Both regions have strong  OA intensity. However, only the  OA spectrum of labelled cells fits the absorbance spectrum of GNRs, which allows distinguishing the GNR-labelled cells from any other region with a high endogenous  OA signal. MSOT imaging of the cell clusters 3 days after injection of $2\times 10^4$ and $1\times 10^5$ (**d**) and $2\times 10^5$ GNR-Si35-labeled cells. Scale bars are 5 mm. **e** The left column shows a single wavelength maximum intensity projection in the xy plane of the regions of interest. The great anatomical resolution of MSOT is observed here, allowing visualization of small vessels (e.g., renal artery and renal vein). The middle column shows the same regions after multispectral processing, enabling high sensitivity detection of GNR-Si35-labeled cells. Volume views of the regions of interest are shown in the right column. Scale bars are 5 mm. Colour scale range is 0 to $1.1\times 10^5$ MSOT au. Reproduced with permission from (https://pubs.acs.org/doi/10.1021/acsnano.6b03246) [38].Copyright ©2016, American Chemical Society. All rights reserved. Further permission related to the material excerpted should be directed to the ACS

Silica coating is an alternative approach in enhancing the cellular uptake of contrast agents like gold nanoparticles. Not only is silica biocompatible, but it also has added benefits of preventing the aggregation of particles in cells. Jokerst et al. reported real-time and non-invasive imaging of a guided stem cell therapy for muscular dystrophy using silica-coated gold nanorods (SiGNRs) [37]. It was observed that the silica coating facilitated the cellular uptake which led to a fivefold signal increase in MSCs loaded with SiGNRs than GNRs, in turn increasing the OA signal by fourfold. Comenge et al. used silica-coated gold nanorods for MSOT [38]. They observed that the plasmon coupling and steric hindrance were avoided upon endocytosis by MSCs due to the silica coating. The optical signature of GNR-Si35 (GNRs with a silica shell thickness of 35 nm) was still preserved after cellular uptake (Fig. 4a, b). The authors were able to monitor $2 \times 10^4$ cells labelled with 30 pM GNRs for 15 days using MSOT (Fig. 4c–e) and they observed that the MSOT in vivo signal intensity decreased with time with cell division reducing the number of GNRs per cell.

Nanoparticles can be optimised with labelling enhancers like CPPs and surface coatings to promote cellular uptake and retention. To image stem cells with high sensitivity and contrast, there is a need to label MSCs with an optimal concentration of the contrast agent without affecting the cell’s normal function.

### Long-term longitudinal monitoring

Long-term longitudinal monitoring of stem cells in vivo is imperative to understand the regenerative pathway of stem cells. Longitudinal studies can be particularly useful for evaluating the development of a regenerative disease, biodistribution of stem cells after transplantation and the outcomes of treatments over time. Unlike photon-emitting contrast agents, optoacoustic contrast agents can be optimised for long-term longitudinal monitoring of stem cells, as their signal does not depend on radioactive decay. Easy renal clearance is possible with nanoparticles smaller than 5.5 nm but that limits their long-term longitudinal capabilities [39].

Modifying the surfaces of inorganic nanoparticles can increase their circulation time in blood and increase their water solubility. Citrate-coated Prussian blue particles (PBPs) were used by Li to monitor traumatic brain injury (TBI) and track the progress of disease recovery [40]. The real-time imaging capability of OAI along with the long-term retention of PBPs enables the monitoring of the damaged vasculature with resulting haemorrhage, the rehabilitation process, and the clearance of the blood clot. Cell labelling stability experiments showed less than 10% PBP leakage post stem cell labelling proving their capability for longitudinal monitoring. 3D model systems mimicking the extracellular matrix (ECM) have enabled monitoring MSC migration and proliferation. 3D ECM’s in vitro microenvironment can potentially enable improved assessment of in vivo cell behaviour in comparison to 2D cell monolayers. 3D ECMs like hydrogels mimic the native tissue microenvironment and thus promote cell proliferation, migration and differentiation [41]. Moreover, encapsulation of the cells in fibrin gels facilitates signal quantification and localised monitoring. Longitudinal imaging of stem cells labelled with gold nano tracers (Au NTs) were evaluated using PEGylated fibrin gel systems. Spectral information from the multi-wavelength optoacoustic imaging allowed imaging at a penetration depth of 10–50 mm for 10 days while the labelled stem cells were intact in the gel [42]. MSC migration and proliferation were temporally assessed in vivo, with nanoparticle loading decreasing exponentially over time because of exocytosis of the nanoparticles by the cells as well as dilution due to cell division. Chung et al. observed that although the PEGylated fibrin system along with the long-term retention capabilities of GNTs enabled stem cell tracking for 16 days, the tracers excreted from the cell may bind to the gel matrix, generating false positives in the OA signal [43].

Rapid clearance of contrast agents from the cell prohibits long-term monitoring and longitudinal studies of stem cells. Developing contrast agents with physicochemical characteristics that enable them to be retained in the cells and delivering labelled stem cells using 3D model systems can facilitate stem cell tracking for longer periods of time. Depending on the retention capability and colloidal stability of the contrast agents used for stem cell labelling, OAI can efficiently track the stem cells in real-time at the time of delivery and can provide longitudinal monitoring after transplantation as long as the signal from the contrast agent is above the detection threshold. In pre-clinical studies, researchers have reported two weeks of longitudinal monitoring of stem cells using nanoparticles as contrast agents [32, 38, 40, 44]. Zhang et al. reported the use of a dual modal contrast agent for the longitudinal tracking of stem cells using OAI for 35 days [45]. Biodistribution studies using mouse models can be carried out before therapeutic studies to assess the longitudinal monitoring capability along with the migration of the labelled stem cells after their implantation. Histological analysis of tissues and organs after biodistribution studies often aid in understanding the delivery and homing of transplanted stem cells and allows for the investigation of abnormalities in the tissue, if any, at different time-points. This can further help in understanding the regenerative mechanism and pathway of stem cells in a disease model for clinical applications.

### Imaging in the NIR- I and NIR-II windows

The development of exogenous contrast agents that provide high imaging contrast is vital to ensure that the signal does not attenuate drastically during imaging process due to background noise and clutter signals as a result of light scattering from tissues. Labelling cells with higher concentrations of contrast agents will enhance the signal to noise ratio but result in cytotoxicity.

Contrast agents with optical absorption in the near-infrared (NIR) wavelengths, where tissue absorption is minimal can be utilised for OAI. With less autofluorescence at NIR compared to visible wavelengths, NIR dyes can be used as optoacoustic contrast agents. For example, 1,1’-dioctadecyl-3,3,3’,3’tetramethylindotricarbo-cyanine-iodide (DiR) was used to label bone-marrow- derived MSCs by Berninger et al. for cell-based cardiac regenerative therapy tracking using MSOT [46]. Due to the high emission at 782 nm compared to other lipophilic membrane dyes, DiR has a higher detection sensitivity in the deep tissues of organs such as the myocardium [47]. Using 10 $\upmu$g/ml DiR, as low as $2\times 10^4$ was detectable in the rabbit heart by MSOT. The red-shifted DiR cyanine derivatives ensured a low fluorescent yield, allowing most of the absorbed light energy to contribute optoacoustic signal generation. The poor hydrophilicity and photostability of conventional NIR organic dyes could be overcome by developing nanostructures that have a localized Surface Plasmon Resonance (SPR) absorption in the NIR region. The optical properties of nanoparticles depend on the property of SPR, whereby an incident electromagnetic field (EM) of a specific frequency induces the resonant oscillation of conduction band electrons on the metal’s surface [48]. The resonant photons are confined by the nanoparticle and this results in an increase in the EM field, thus enhancing their optical properties. Gold nanoparticles can be easily synthesized into a variety of shapes and sizes, thus can be utilised to influence the nature of the SPR and can be tuned to absorb in the NIR region.

Varying the aspect ratio or changing the dimensions can help tune the relative scattering to absorption contribution of nanoparticles. Stem cell delivery to a trabecular meshwork (TM), a fluid drainage tissue in the anterior eye was studied using gold nanosphere (AuNS) labelled MSCs by Kubelick et al. [49]. Reduced cellularity in the TM can often elevate intraocular pressure, one of the main causes of glaucoma. Although the absorption peak of AuNS is 520 nm, the AuNS-labelled MSCs have an absorption peak at 700 nm. This redshift is a result of surface plasmon resonance coupling upon nanoparticle endocytosis enabling tracking of stem cell migration in the anterior segment of the eye [50]. OAI in conjunction with a suitable contrast agent can improve weakly scattering structure, imaging depth, and can potentially detect molecular activity.

When anisotropy is introduced to nanoparticles, the SPR peak can be strongly enhanced and tuned as a function of aspect ratio. Anisotropic nanoparticles, due to their versatile geometries have large extinction cross-sections. This ensures that high optical absorption in the NIR region where deep-tissue imaging with a high signal-to-noise ratio (SNR) is possible. Quantitative tracking of human MSCs labelled with Gold Nanocages (AuNCs) used the strong optical absorption and two- and three-photon luminescence properties of AuNCs [51]. The hollow interiors and the porous walls of these nanostructures enable transport and delivery of bioactive substances in the body and improved detection sensitivity due to their large optical absorption cross-section [52].

Photon absorption and scattering in biological tissue is characterized by many factors such as cell size, water content, electrolyte concentration and blood oxygenation. Absorption and scattering also depends on the wavelength range and is minimal around 1300 nm and 1700 nm [53].The laser setup for NIR-I (650 to 950 nm) imaging is expensive, bulky and inefficient when compared to NIR-II imaging. Due to low scattering and absorption from biological tissues and the availability of lasers with the required pulse duration, repetition rate and energy required for OAI, imaging at 1064 nm is of particular interest [21]. The maximum permissible exposure (MPE) is the maximum electromagnetic energy that can be disposed in a particular volume of a biological matter before denaturation or damage to the tissue will occur. As the MPE depends on the wavelength and the number of photons, it will also limit the photon count that is statistically detectable from scattering structures in deeper tissue regions. MPE at 1064 nm is about 1000 mW/cm$^2$ per pulse according to the American National Standards Institute (ANSI) safety limit, which results in more photons and increased probability of detecting light from deeper regions compared to 300 mW/cm$^2$ MPE at 800 nm [54]. Dual plasmonic gold nanostars with absorption maxima at 1064 nm were designed and synthesized as optoacoustic contrast agents to utilize these advantages [55]. For longer wavelengths in the NIR-II window, fivefold higher MPE allows deep-tissue imaging compared to wavelengths in the visible windows [56]. Since water has a strong absorption around 1400–1500 nm, exogenous contrast agents with an absorption peak in this range would not be ideal for deep tissue imaging.

**Fig. 5:**
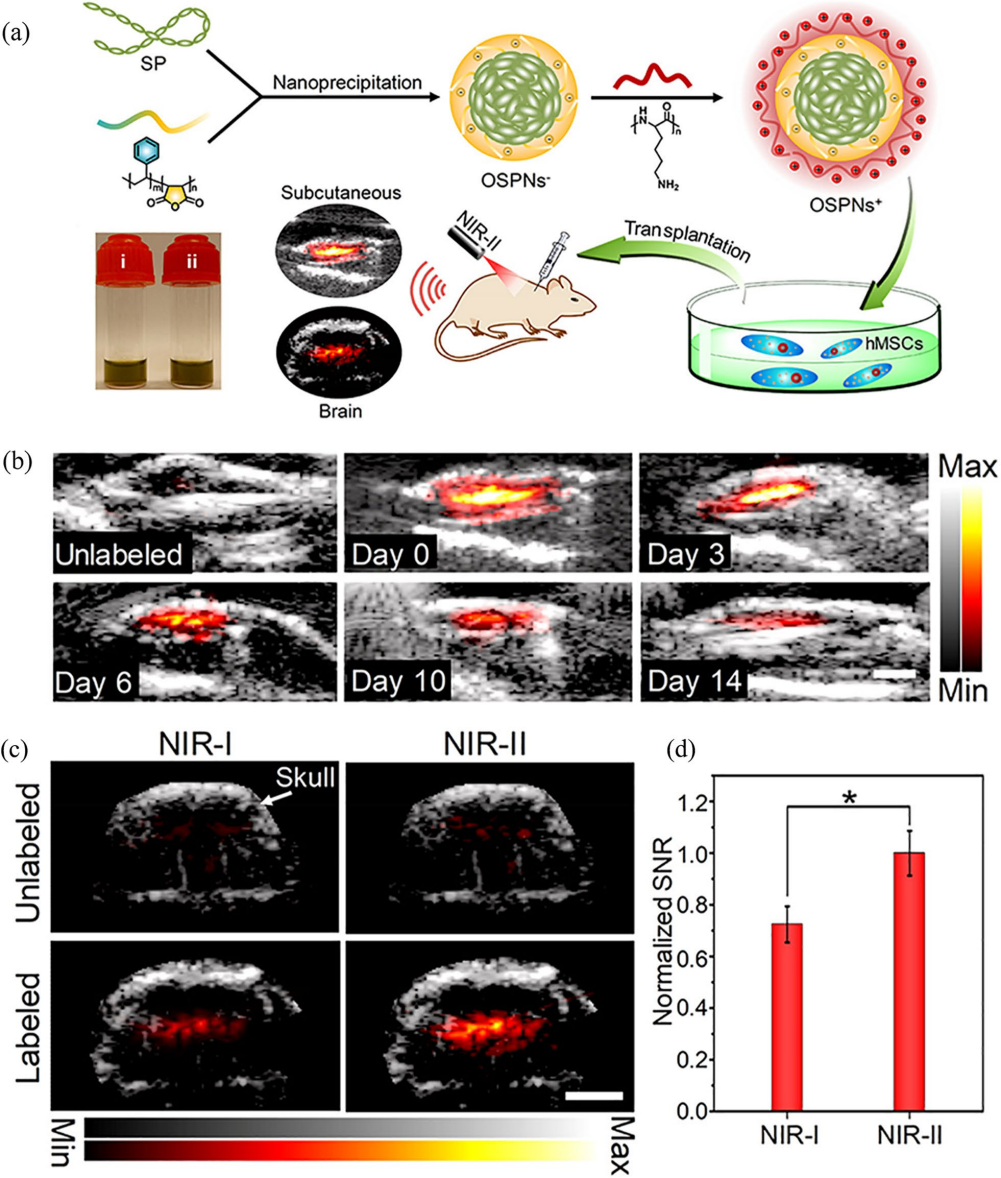
**a** Illustration of the preparation procedure of OSPNs$^+$ and the  OA labelling of hMSCs after transplantation. **b** Merged ultrasound (US, gray scale) and OA images of subcutaneously transplanted unlabelled or OSPNs$^+$- labelled hMSCs (10$^5$ cells) in vivo. Images of OSPNs$^+$- labelled cells were acquired serially at different post-injection time (days 0, 3, 6, 10, and 14). OA signals were recorded at 1064 nm (energy density: 5 mJ/cm$^2$). Scale bar: 3 mm. **c** Combined US and OA imaging of unlabelled or OSPNs$^+$- labelled hMSCs (80$\times$10$^3$ cells) injected into the brain of nude mice using NIR-I (860 nm) or NIRII (1064 nm) light excitation. The energy density of the laser was adjusted to the same value at 10 mJ/cm$^2$. Images were collected immediately after cell injection. Arrow indicates the skull position. Scale bar: 3 mm. **d** Normalized OA SNR of OSPNs$^+$- labelled hMSCs implanted into mice brain under NIR-I (860 nm) or NIR-II (1064 nm) light excitation ($^*$p < 0.05). Adapted with permission from [44].Copyright $\copyright$ 2018, American Chemical Society

Designing exogenous contrast agents in the NIR-II window include challenges such as favourable clearance profiles, biocompatibility and photothermal conversion efficiency of NIR-II contrast agents similar to clinically approved NIR-I contrast agents. NIR-II contrast agents tend to have complex geometrical structures leading to production issues such as reproducibility and homogeneity. Organic semiconducting polymer nanoparticle probes (OSPN$^+$) were used by Yin et al. as an exogenous contrast agent in the second NIR window for stem cell tracking using optoacoustic imaging [44]. OSPN$^+$ have a hydrophobic semiconducting polymer core (SP), an amphiphilic polystyrene maleic anhydride (PSMA) and a Poly-L-lysine (PLL) coating (Fig. 5a). The SP cores are responsible for the OA signal and OSPN$^+$ have abroad absorption spectrum covering both NIR-I and II with absorption peaks at 916 and 1025 nm. As optical scattering from surrounding tissues is lower at longer wavelengths, a higher SNR was observed in the NIR-II window compared to NIR-I at the same concentration and imaging depth (Fig. 5b–d) [57–59].

Fluorescent dyes like DiR which have low quantum yields and anisotropic nanostructures with well-controlled shapes and sizes, are preferred for OAI. In comparison to the first NIR window, the NIR-II window offers high contrast imaging, better penetration depth and higher MPE. Due to the minimal tissue absorption and scattering in the second NIR region, more research is needed to engineer NIR-II contrast agents to improve depth of imaging. Also, efficient cellular uptake and pharmacokinetics of NIR-II contrast agents must be studied as they possess complex geometries.

### Stem cell viability detection

Monitoring stem cell distribution, function and survival are critical for therapeutic translation to patients. However, most contrast agents cannot distinguish live cells participating in the regeneration process from dead cells, leading to false positive signals and errors in quantification during imaging. The optimal contrast agent should emit a positive signal for live labelled cells only. However, that is not always the case. Measuring changes in optoacoustic properties associated with viability and/or functionality of stem cells e.g., enzymatic activities, growth factors, chemicals secreted during cell differentiation, changes in intracellular and extracellular pH, differences in metal ion levels and the production of reactive oxygen species (ROS) upon cell death could act as stimuli for detection.

**Fig. 6:**
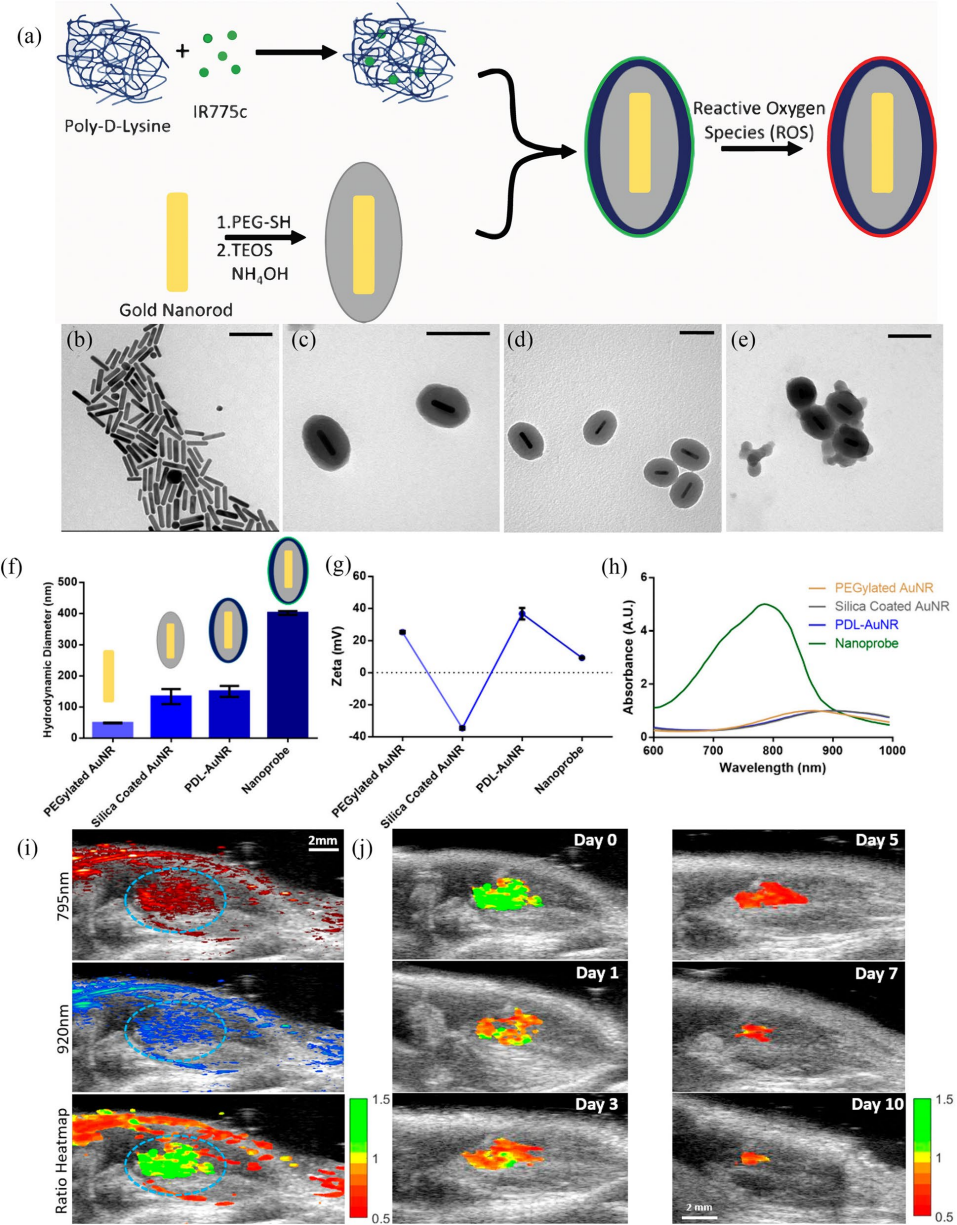
**a** Diagram of nanoparticle synthesis. The IR775c/PDL is electrostatically bound to the silica coated AuNRs. The particle is composed of the polymer/dye mixture on the outside of the AuNR (green), allowing for ROS to interact with the dye and degrade it (red), while the inert AuNR does not change, thus giving different photoacoustic signals due to ROS interaction. TEM images of **b** gold nanorods, **c** silica-coated AuNRs, **d** PDL layered silica-AuNRs, and **e** PDL/IR775c layered silica-AuNR were obtained. Scale bar is equal to 100 nm. **f** The hydrodynamic diameter increased for each step with the final nanoparticle having a diameter of 400 nm. **g** The $\zeta$-potential for each step was collected displaying flipping $\zeta$-potential with each layer and a relatively neutral $\zeta$-potential for the final particle. **h** The absorbance spectra displayed changing peak absorbance from 875 nm for AuNR to 910 nm for silica–AuNR and PDL-coated silica–AuNR, and 780 nm for the final nanoparticle. Scale bar is equal to 100 nm. **i** Imaging stem cells in vivo. The US/OA image of transplanted stem cells on day 0 is visualized at 795 and 920 nm excitation, correlating with the IR775c dye and AuNR peak. The labelled stem cells are circled in blue. The last image is a heatmap using the ratio of 795 nm/920 nm. The stem cells display a high ratio as expected due to them being alive directly after transplantation. **j** Ratiometric imaging of stem cell viability in vivo. OA images of day 0, 1, 3, 5, 7, and 10 at 795 and 920 nm were obtained, and the ratiometric images were created. The surrounding tissue signal was subtracted to better visualize the change in stem cell viability during the study. Reduction in ratio and signal is visible. Adapted with permission from [61].Copyright $\copyright$ 2019, American Chemical Society

Viability of stem cells was monitored by Yoo et al. using ICG due to its’ relatively high optical absorption at 800 nm (NIR) and its ability to clear out of stem cells after cellular death through diffusion [60]. Continuous assessment of the amount of viable transplanted cells over time is crucial for developing effective stem cell therapies in a clinical setting. Since OAI enables simultaneous imaging of endogenous and exogenous contrast agents, dynamic biological processes such as apoptosis of stem cells after transplantation be visualized using activatable contrast agents for a continuous period. Upon cell death, these smart contrast agents induce a shift in the peak absorption wavelength and generate a prescribed signal change, thus enabling OAI to track the survival of stem cells up during the therapy. This was achieved by Dhada et al. by coating gold nanorods with IR-775 chloride (IR775c), which is a reactive oxygen species (ROS) sensitive NIR dye for assessing in vivo MSC viability (Fig. 6a) [61]. This nanoprobe emits at two peaks (IR775c from 780–800 nm and a smaller peak at 910 nm associated with the AuNR coating) (Fig. 6b-h). However, during in-vivo optoacoustic imaging, it was observed from the optoacoustic spectrum that the two absorption peaks of the nanoprobe were red shifted to 795 nm and 920 nm respectively because of optical attenuation and scattering by the surrounding tissue. A ratiometric analysis of the dye/nanorod peaks was utilised to monitor the stem cell viability as with cell death, the ROS generated by the apoptotic stem cell degrades the IR775c dye causing a reduction in its peak signal with no effect on the spectral shape or peak signal intensity of AuNR (Fig. 6i–j). However, this sensing mechanism of the nanoprobe can only monitor the viability of labelled stem cells and cannot account for the reduction of sensitivity due to cell proliferation and migration in tissue. Another challenge observed with direct cell labelling using contrast agents is the ability of dead cells to generate a signal in vivo because of their presence in cellular debris or due to uptake by phagocytic cells. Ricles et al. engineered a dual nanoparticle system consisting of PEGylated gold nanospheres (absorption maximum at 520 nm) and PLL coated silica GNRs (optical absorption at NIR (750 nm)), which were taken up by macrophages and MSCs, respectively [62]. Macrophages were observed to endocytose nanospheres at a faster rate than the MSCs due to their characteristic non-specific uptake whereas the positively charged PLL coated silica GNRs were readily endocytosed by MSCs. This in vivo analysis of the preferentially labelled cells enabled assessment of the key roles of macrophages in wound healing and vascular regeneration regeneration [63–66]. Due to the spectroscopic nature of OAI, the dual nanoparticle system assisted the monitoring of synergistic crosstalk between the two cell types while assessing stem cell viability.

False positive signals can originate from the contrast agent either trapped in dead cells or engulfed by macrophages could yield incorrect results during in vivo stem cell tracking. Dyes like ICG diffuses out if the labelled cell undergoes apoptosis. Contrast agents that can detect ROS generation or a change in pH can be engineered for real-time in vivo monitoring of stem cell viability.

## Multimodal optoacoustic contrast agents

As combining imaging modalities is advantageous in extracting detailed morphological and molecular information, a huge array of complex multimodal exogenous contrast agents is being developed. This exercise will reduce the potential drawbacks of using each imaging modality alone and modality-specific strengths can be integrated to form a more complete picture of each transplantation. While selecting imaging modalities for stem cell tracking, it is important that the multimodal imaging contributes to relevant and specific information during stem cell transplantation such as stem cell localisation, viability, quantification, and functionality. Although multimodal imaging systems can yield more information per sample or subject, it is important to consider the number of imaging sessions required, imaging time, cost and repeated or frequent anaesthetic use in pre-clinical imaging studies. Moreover, false positive or false negative results could be cross-checked using multimodal imaging. Imaging techniques utilizing multimodal contrast agents can provide more insight as they can easily compensate for limitations from a single imaging modality.

**Fig. 7:**
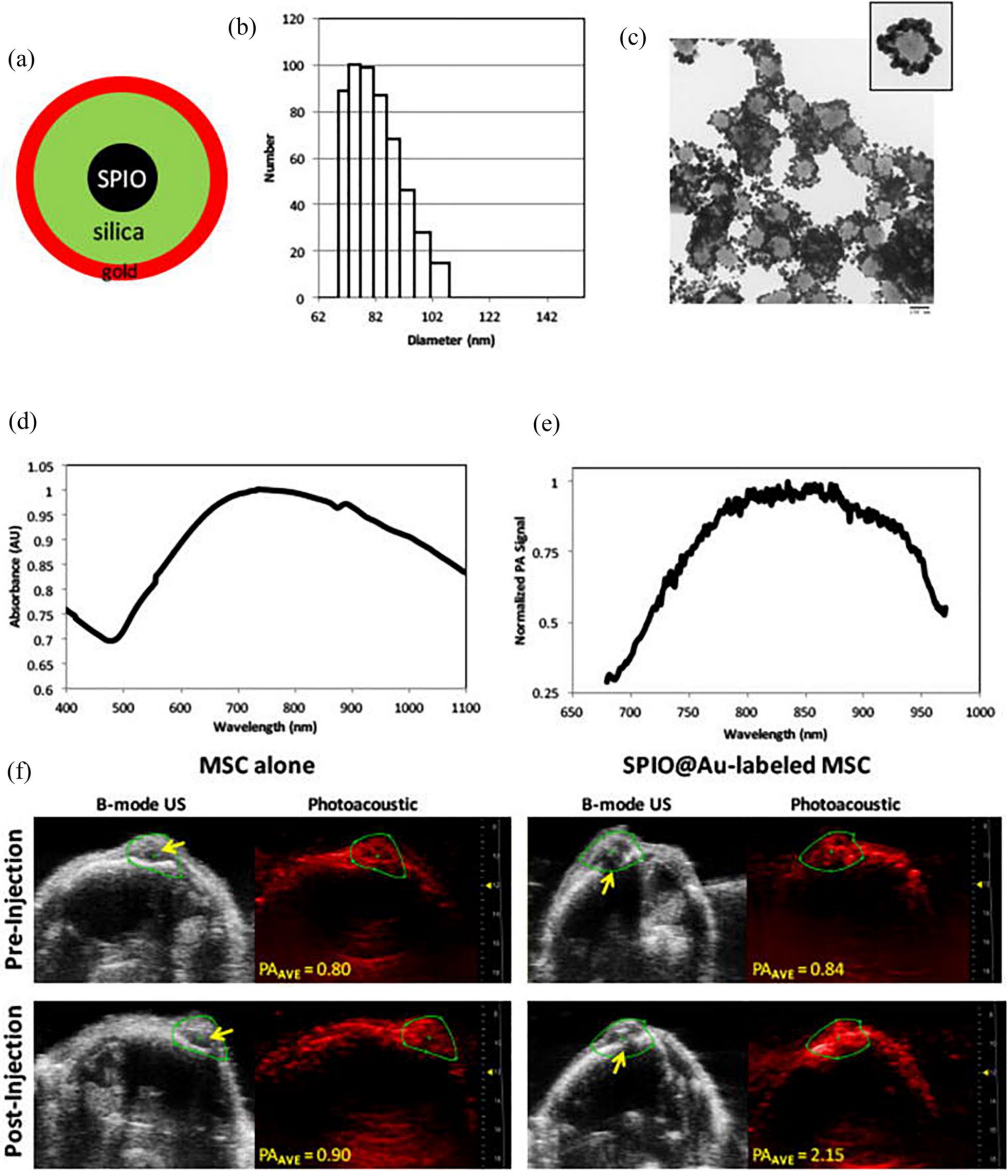
**a** Schematic illustration of SPIO@Au nanoparticle containing SPIO core, silica, and gold coating. **b** Dynamic light scattering analysis and **c** transmission electron microscopic image of SPIO@Au indicating the size of the nanoparticle. Scale bar is 100 nm. **d** Ultraviolet-visible light absorption spectrum and **e** optoacoustic (OA) profile of SPIO@Au. **f** In vivo OA imaging of mouse brain at 72 h after intra-carotid artery injection of SPIO@Au-labelled MSCs or MSCs alone. Yellow arrows on B-mode ultrasound images of the brain indicate the bolt placement where the U87 cells were implanted into the brain. The OA images were taken at 810 nm, and the signal intensity was calculated within the green regions of interest. Average OA values were obtained by averaging all PA intensity values above the signal-noise threshold within the regions of interest. Reproduced with permission from [67].Copyright $\copyright$ 2018, IOP Publishing. All rights reserved

Nanoparticles are ideal as multimodal contrast agents as they have large surface areas that can be easily functionalised with different components to support multimodal imaging. Different molecular components can also be encapsulated or embedded into single nanoparticles. The molecular constituents can be attached to the carrier nanoparticle by electrostatic forces, covalent or conjugated bonding, and can allow the incorporation of chemicals into the nanoparticle core. Magnetic resonance imaging (MRI) is a well-established imaging modality for stem-cell tracking as it provides detailed morphological and functional information and allows high resolution and accurate localization of cells. MRI contrast agents can be classified based on their chemical composition, magnetic properties, the presence or absence of metal atoms, effects on MR images and their biodistribution. However, not all patients can be imaged using this expensive and complex procedure. Also, MRI’s limited sensitivity and inability to quantify the cell population can be compensated using OAI. Human MSCs labelled with multifunctional nanoparticles containing superparamagnetic iron oxide with a gold coating (SPIO@Au), which have a maximum absorbance at 810 nm, were used by Qiao et al. for imaging brain tumours (Fig. 7a–f) [67]. The core-shell structure of the SPIO@Au contrast agent is the source of its dual-modal activity. The localisation of 10$^6$ labelled MSCs into a tumour was exhibited by progressive hypo-intensity of the tumour visible in the MRI signal after enhancement with optoacoustic imaging for up to 3 days (Fig. 7f). The core-shell structure of the SPIO@Au contrast agent is the source of its dual-modal activity. They demonstrated that OAI can be used in conjunction with MRI and confirmed the delivery of MSCs into the brain via intra-carotid artery injection. Designing multimodal contrast agents requires optimisation of the concentration ratios of the respective contrast materials to ensure high detection sensitivity for all imaging modalities. Lemaster et al. reported the use of synthetic melanin nanoparticles loaded with gadolinium (Gd (III)-SMNPs) as a dual modality contrast agent for OAI and MRI [68]. The optimized concentrations of the SMNP and the Gd (III) in the dual MRI/OA contrast agent along with the improved absorption cross-section due to the melanin coordination of the metal ions via catechol groups influence the optoacoustic signal enhancement [69]. This chelation not only increased the biocompatibility of Gd-based contrast agents but also showed a 64-fold increase in the OA signal intensity and a twofold increase in the MRI signal contrast in vivo. The dual-modal polyethylene glycol-modified magnetic nanoparticles (Fe$^{3+}$-PEG-MNP) based on natural biomaterials including melanin and Fe ions for OAI and MRI of bone marrow (BM)-derived MSCs developed by Zhang et al. are perfect candidates for clinical stem cell therapies [45]. Their ultra-small particle size (9.2 nm) and water solubility make them highly stable while enhancing cellular labelling and low cell cytotoxicity. Their neutral surface charge enabled labelling of 98.56% BM-MSCs and facilitated 35 days of OAI and 28 days of MRI in vivo.

Ultrasound imaging (USI) and OAI can be combined to provide guidance for accurate stem cell transplantation. Exact co-registration of USI and OA images can be attained by using the same transducers for detection. The limited contrast and 3D capabilities of USI can be compensated with OAI. USI in turn can assist OAI by providing structural and morphological tissue information. This aids the acquisition of information regarding the position and depth of the labelled stem cells. Donnelly et al. used the hybrid OA/US imaging system to guide the delivery of plasmonic gold nanosphere (AuNS)-labelled MSCs to the spinal cord for treating neurodegenerative diseases and traumatic injuries [70]. This real-time optoacoustic imaging technique combined with ultrasound minimized the risk of needle shear and enabled the correct targeting of the injection, thus improving therapeutic outcomes [71, 72]. More specifically, the ultrasound provided images of the patient’s surface vasculature, which aided the identification of oxygenated and deoxygenated blood using OAI. Spectroscopic OAI facilitated in real-time tracking the migration of the transplanted stem cells labelled with AuNSs and allowed in distinguishing the endogenous contrast of the white matter from the grey matter in the ventral portion of the spinal cord at 1730 nm. Lemaster et al. developed a poly (lactic-co-glycolic acid) (PLGA)-based iron oxide nanobubble labelled with DiR as a trimodal contrast agent. The PLGA coating facilitated USI and the DiR enhanced the OA signal. Moreover, the iron oxide increased the magnetic particle imaging (MPI) signal for tracking cardiac stem cells [73]. Although USI provides a high temporal resolution (300 Hz), OAI can achieve a better contrast for structures in soft tissue. Moreover, the imaging depth can be improved with MPI even if it only provides low temporal resolution. While MPI has the same limitations as MRI, MPI provides a high contrast-to-noise ratio. The multifunctionality of this hybrid nanobubble overcomes the limitations of each modality.

For stem cell therapy, monitoring different stages of cell migration and progress of cell differentiation may be monitored using multimodal imaging methods. To decide which imaging modalities are best suited for specific applications, it is vital to consider what relevant and specific information each imaging modality can provide. Once the imaging modalities are chosen, the careful design of the contrast agent is an essential step for multimodal detection.

## OAI contrast agents for stem cell tracking applications in the clinic

Stem cell therapies are currently being investigated for cardiovascular diseases such as ischemic heart disease, glaucoma, neurological diseases such as cerebral ischemia, Parkinson’s disease, liver diseases and degenerative musculoskeletal disorders such as osteoarthritis. Thus, it is imperative to optimize the delivery of stem cells and improve the efficacy of these therapies by using exogenous contrast agents in future clinical trials.

One of the most challenging issues for successful stem cell therapy is the efficient delivery of stem cells to the tissue of interest without damaging the host tissue. The real-time imaging capabilities of OAI can aid the physician in the clinic regarding the delivery of labelled stem cells to the implantation site. OAI can thus overcome this limitation of MRI by providing real-time guidance during transplantation. Yao et al. developed a trimodal theragnostic probe that increased efficacy of stem cell therapy of ischemic stroke and used OAI to guide intracerebral injection [74]. Kubelick and Emelianov also reported the use of OAI for the real-time guidance of stem cell injection in the spinal cord in rodents [75].

OAI demonstrates an immense potential for clinical applications of stem cell tracking due to its ability to provide structural, molecular, functional and kinetic information from endogenous contrast agents like hemoglobin, melanin, lipid and water in addition to labelled cells. OAI can differentiate between oxygenated and deoxygenated blood and allows monitoring of blood oxygenation saturation, thereby making it an attractive imaging technique for stem cell therapies for ischemic diseases.

Along with stem cell tracking, OAI also offers immense potential for musculoskeletal diseases due to its additional capacity to identify and characterize soft-tissue inflammation based on the detection of hemodynamic changes. Early detection and grading of inflammation can be beneficial for arthritic patients, as they could be advised to consider a more targeted treatment regime or timely stem cell therapy treatments or to improve their quality of life, for example. Moreover, non-responders could be identified, and treatment modifications could be implemented earlier on a case-by-case basis.

The detection and therapeutic monitoring of breast cancer has been a primary focus for employing OAI in the clinic and this has been reviewed in detail [76]. As stem cells can be employed as potential therapeutic carriers, Xu et al. developed an MSC delivery system for OAI imaging and chemo-photothermal therapy of triple negative breast cancer. This multifunctional contrast agent was composed of lipids, GNRs, iron oxide nanoclusters and doxorubicin (DOX), a chemotherapy agent. Upon light irradiation, a controlled release of DOX facilitated efficient therapeutic response along with photothermal therapy using GNRs [77].

The main limitations for translating stem cell tracking to the clinic are long term cytotoxic effects of contrast agents, signal dilution as a result of cell proliferation, the insufficient capability of monitoring the viability and functionality of labelled stem cells in vivo and the possibility of uptake of contrast agents by macrophages following stem cell apoptosis. Focusing efforts on the development of biocompatible and multifunctional OAI contrast agents can lead to their rapid clinical translation for stem cell therapy applications. Fundamental information regarding dose optimization, engraftment site preference and the timely delivery of stem cells could be evaluated during long-term in vivo preclinical experiments and can tailor stem cell treatments for individual patients. Moreover, the OAI technology can be used in conjunction with US and MRI as it demonstrates excellent capacity to guide and provide additional information for improving stem cell therapies and allows personal patient management in the clinic.

## Conclusion

Engineering suitable exogenous contrast agents for stem cell tracking is essential to perform and evaluate therapeutic stem cell delivery and retention in clinical applications. For safe and efficient stem cell therapeutic practices, the ability to monitor the biodistribution of cells and to determine their survival rate after transplantation is an important goal. Furthermore, suitable contrast agents allow monitoring of long-term effects of stem cell transplantation, such as the host immune response and undesired proliferation of implanted cells. At present, there is no imaging modality that can monitor all aspects of therapeutic intervention using stem cells in the clinical setting.

While MRI and other imaging modalities have proven successful in tracking stem cells, OAI is of particular interest as a hybrid imaging modality that combines the high contrast of optical imaging with the large depth of ultrasound imaging. Stem cells can be imaged using OAI by labelling them with exogenous contrast agents. The emission and absorption properties of contrast agents used for labelling stem cells allow the extraction of additional molecular information within the imaging voxels. Colloidal stability and biocompatibility of contrast agents reduces the labelled cell’s probability of being detected as a foreign substance in the body after transplantation. Molecular probes that can be easily surface modified for bio-conjugation should be engineered to improve target specificity and long-term stability. The size and geometry of the contrast agent along with its surface charge and functionalization could result in protein adsorption or formation of a protein corona onto the surface of the contrast agent, which in turn could affect its cellular uptake.

For OAI, contrast agents that have a high light-to-ultrasound conversion efficiency and a high optical absorption in the NIR optical window are highly advantageous for deeper penetration into biological tissue. Optimal imaging depth can be achieved at 730 nm, or at 1064 nm, which is less sensitive to blood and melanin concentration. A further advantage of the latter is that the MPE for skin is 100 mJ/cm$^2$ for 1064 nm laser, compared to 25 mJ/cm$^2$ for 750 nm laser. Real time detection of stem cell viability is crucial in a clinical setting as it gives information about the survival rate of stem cells upon engraftment. Conjugation of these contrast agents with various molecular probes can facilitate targeted delivery and the development of multimodal contrast agents. In summary, an ideal exogenous contrast agent for stem cell tracking is one that:

- Is biocompatible and colloidally stable
- Allows easy surface modification
- Enhances cellular uptake
- Allows long-term longitudinal monitoring
- Allows imaging in the second near infrared window (1000–1400 nm)
- Can detect stem cell viability in real time
- Can facilitate multimodal imaging

For future stem cell tracking applications, multimodal and multifunctional contrast agents are an important research area.

## Data Availability

Please contact author for data requests.

## Abbreviations

OAI: Optoacoustic imaging
OA: Optoacoustic
MRI: Magnetic resonance imaging
PAI: Photoacoustic imaging
PA: Photoacoustic
MSOT: Multispectral optoacoustic tomography
NIR: Near-infrared
SNR: Signal to noise ratio
MSCs: Mesenchymal stem cells
ICG: Indocyanine green
FDA: Food and drug administration
PEG: Poly-ethylene-glycol
PLL: Poly-L-lysine
PBNP: Prussian blue nanoparticles
RESV: Resveratrol
ROI: Region of Interest
CPPs: Cell Penetrating peptides
PANPs: Photoacoustic nanoparticles
hESC-CMs: Human embryonic stem-cell-derived cardiomyocytes
GNRs/AuNRs: Gold nanorods
SiGNRs: Silica coated gold nanorods
GNR-Si35: GNRS with a silica shell thickness of 35 nm
PBPs: Prussian blue particles
TBI: Traumatic brain injury
ECM: Extracellular matrix
Au NTs/GNTs: Gold nanotracers
DiR: 1,1’-dioctadecyl-3,3,3’,3’-tetramethylindotricarbo-cyanine-iodide
SPR: Surface plasmon resonance
TM: Trabecular meshwork
AuNS: Gold nanospheres
AuNCs: Gold nanocages
MPE: Maximum permissible exposure
ANSI: American National Standards Institute
OSPN: Organic semiconducting polymer nanoparticle probes
SP: Semiconducting polymer core
PSMA: Polystyrene maleic anhydride
ROS: Reactive oxygen species
PLGA: Poly (lactic-co-glycolic) acid
SPIO@Au: Superparamagnetic iron oxide with a gold coating
Gd(III)-SMNPs: Synthetic melanin nanoparticles doped with gadolinium
Fe3+-PEG-MNP: Polyethylene glycol-modified magnetic nanoparticle
BM-MSCs: Bone mesenchymal stem cells
USI: Ultrasound imaging
MPI: Magnetic particle imaging
